# A Quantum Annealing Approach for Solving Optimal Feature Selection and Next Release Problems

**DOI:** 10.3390/e28080886

**Published:** 2026-08-06

**Authors:** Yuxuan Zhang, Shuchang Wang, Wei Yang

**Affiliations:** 1 School of Computer Science and Technology, University of Science and Technology of China, Hefei 230026, China; yxzhang123@mail.ustc.edu.cn (Y.Z.);; 2 Hefei National Laboratory, University of Science and Technology of China, Hefei 230088, China

**Keywords:** next release problem, feature selection problem, multi-objective binary optimization, quantum annealing

## Abstract

Search-based software engineering (SBSE) tackles critical optimization problems in software engineering, including the next release problem (NRP) and feature selection problem (FSP). Traditional heuristic approaches and integer linear programming (ILP) methods work well for small- to medium-scale problems but face growing computational cost as instances scale up. We investigate quantum annealing (QA) as an optimization subroutine for multi-objective SBSE problems. We propose two QA-based algorithms tailored to different problem scales. For small-scale problems, we reformulate multi-objective optimization (MOO) as single-objective optimization (SOO) using penalty-based mappings for quantum processing. For large-scale problems that exceed current hardware capacity, we employ a decomposition strategy guided by maximum energy impact (MEI) that partitions the problem into smaller sub-QUBOs, integrating QA with a steepest-descent method for local search. Applied to NRP and FSP, our approaches are benchmarked against the heuristic NSGA-II, IBEA, and MOEA/D, as well as the ILP-based ϵ-constraint method. The experimental results reveal that while our methods produce fewer non-dominated solutions than ϵ-constraint, they achieve substantial reductions in execution time. Compared to the evolutionary baselines, our methods achieve competitive solution quality with lower runtime on the instances that they can encode. The penalty-based QUBO formulation fails to reach feasible regions on constraint-dense FSP instances, limiting the current applicability of the approach. QA is a promising but still hardware-limited component for multi-objective SBSE workflows, rather than a wholesale replacement for classical solvers.

## 1. Introduction

Software engineering is a vast and multifaceted field, encompassing the development, management, and enhancement of software. These considerations frequently involve striking a balance between different objectives that may compete or clash with one another [1]. A key challenge is the decision-making process in feature prioritization for software, where cost-effectiveness must be balanced with project requirements. Search-based software engineering (SBSE) addresses such challenges by conceptualizing them as search-based optimization problems and employing different kinds of optimal search algorithms derived from operational research to discover feasible solutions. Among the broad applications of SBSE, this work focuses on two fundamental optimization problems in software requirements engineering and software product line engineering: the next release problem (NRP) and the feature selection problem (FSP), both critical to advancing software engineering practices.

Many optimization problems involve multiple competing objectives that require simultaneous optimization, forming what are known as multi-objective optimization problems. Unlike single-objective optimization, which seeks a single optimal solution, these problems yield a set of solutions, collectively forming the Pareto front [2]. Within this set, no solution is universally superior to another; improving one objective often comes at the expense of another. The complexity of navigating the Pareto front has driven extensive research, with numerous methodologies proposed to address its inherent challenges. The Pareto front represents an area of interest in multi-objective optimization studies due to its inherent complexity and the challenges that it presents in achieving optimal solutions.

## 2. Related Work

The NRP and FSP are typical multi-objective optimization problems in software engineering, with numerous schemes proposed for their solution. NRP methods primarily fall into two categories: multi-objective evolutionary algorithm (MOEA) based on heuristic thought, and integer linear programming (ILP) based on operations research knowledge. Early in 2007, NSGA-II was used to solve the multi-objective NRP (MONRP) [3], followed by other evolutionary algorithms, such as genetic algorithms (GAs), ant colony optimization (HACO) with local search [4], particle swarm optimization with greedy initialization [5], and a hybrid artificial bee colony and differential evolution method (HABC-DE) [6]. On the ILP side, the ϵ-constraint method was introduced in 2015 to address the NRP [7], with subsequent improvements like I-EC for bi-objective NRP and SolRep for tri-objective NRP [8].

Similarly, the FSP has also been tackled using both MOEA and ILP methods. The GA was first applied to the FSP in 2011 [9] and showed good performance. The introduction of MOEAs further advanced FSP solutions, with the Improved Binary Evolutionary Algorithm (IBEA) emerging as the most effective MOEA [10]. Subsequent hybrid approaches, such as IBED, which combines IBEA with differential evolution (DE), improved performance further [11]. Other hybrid strategies included integrating IBEA with constraint solvers, leading to the development of SATIBEA and SMTIBEA [12,13]. In addition to heuristic approaches, ILP methods like ϵ-constraint and SolRep [14] have been applied to the FSP. Notably, researchers have also explored combining ILP with IBEA, resulting in the MILPIBEA method [15].

MOEAs exhibit remarkable versatility, as they can solve problems without necessitating specific domain knowledge. However, their efficacy diminishes as the problem size increases, due to a sharp rise in computational time [16]. MOEAs often fail to identify all Pareto solutions, limiting their utility. In contrast, ILP holds the potential to find out all Pareto solutions. Nevertheless, it is not suitable for middle- or large-scale combinatorial optimization problems, which are NP-hard problems. As the problem size expands, the time required for finding valid solutions grows exponentially, making ILP less efficient. Therefore, when dealing with large-scale problems, both MOEAs and ILP grapple with the challenge of finding feasible solutions within a restricted time, hindering their application in complex software engineering environments. This highlights the urgent need for an efficient approach that can deliver high-quality solutions quickly in the context of SBSE.

Recent years have witnessed growing interest in applying quantum and quantum-inspired optimization techniques to combinatorial problems. The Quantum Approximate Optimization Algorithm (QAOA) and Variational Quantum Eigensolver (VQE) have been explored for graph partitioning, scheduling, and constraint satisfaction problems [17,18]. Quantum-inspired evolutionary algorithms incorporate quantum concepts such as superposition into classical metaheuristics [19]. Digital annealers, which simulate quantum annealing on specialized classical hardware, have been deployed for portfolio optimization and traffic routing [20]. While these approaches have demonstrated promise in operations research and industrial optimization, their application to software engineering decision problems—particularly multi-objective release planning and feature selection—remains limited.

Quantum annealing (QA) is a physical optimization technique that exploits quantum tunneling and superposition to explore solution landscapes. Its development can be traced to early quantum computing concepts in the 1980s [21], and commercial QA systems now integrate thousands of qubits. Theoretical work has shown that certain quantum algorithms offer polynomial or exponential speedups over classical methods [22,23], although practical demonstrations of quantum advantage remain confined to specific problem classes and hardware configurations.

**Figure 1:**
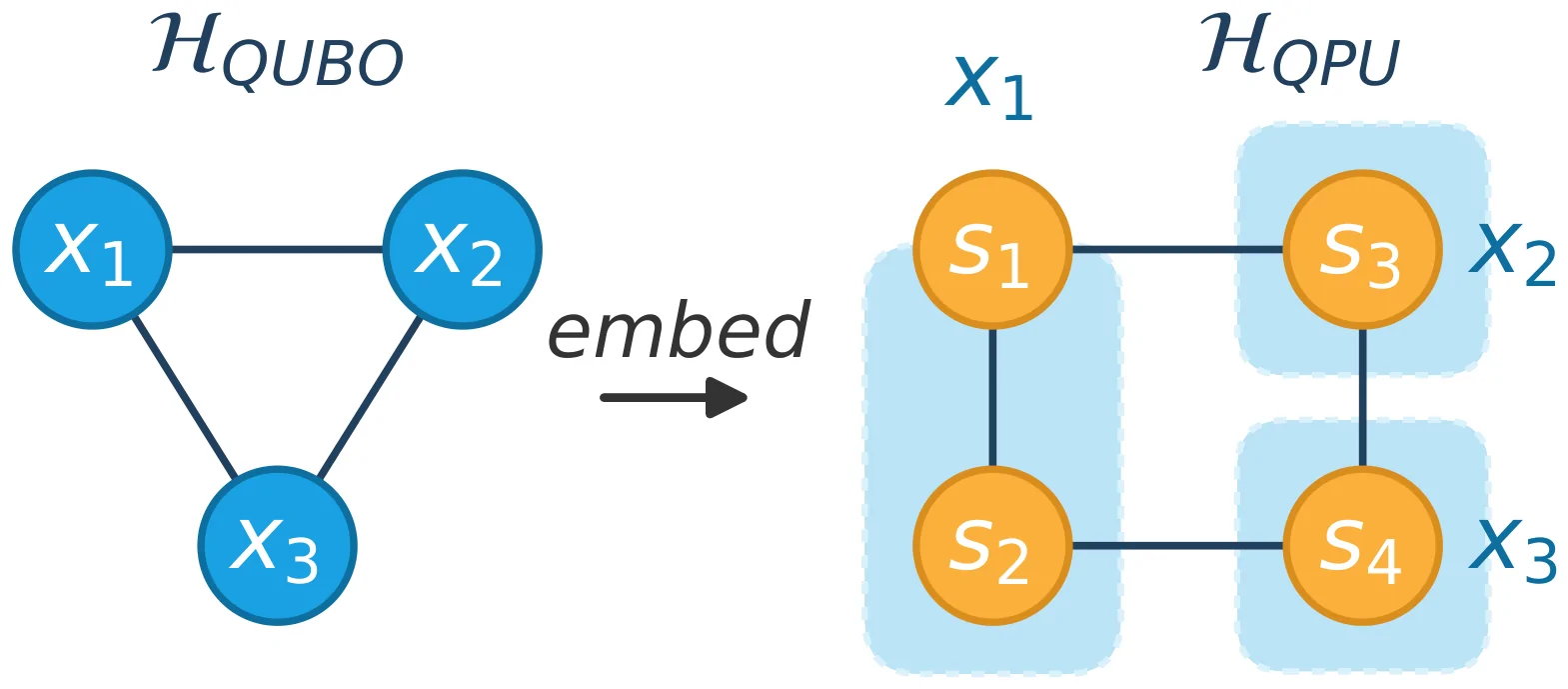
Embedding on a QPU. This figure shows a QUBO problem embedded on a QPU. In the left part of the figure, $H_{QUBO}$ has linear terms $x_{1}$, $x_{2}$, and $x_{3}$ and quadratic terms $x_{1}x_{2}$, $x_{1}x_{3}$, and $x_{2}x_{3}$. In the right part of the figure, $H_{QPU}$ is described by qubits $s_{1}$, $s_{2}$, $s_{3}$, and $s_{4}$ and couplers $s_{1}s_{2}$, $s_{1}s_{3}$, $s_{2}s_{4}$, and $s_{3}s_{4}$; $x_{1}$ is mapped to $\left\{s_{1},s_{2}\right\}$, while $x_{2}$ and $x_{3}$ are mapped to $s_{3}$ and $s_{4}$, respectively; $\left\{s_{1},s_{2}\right\}$ is called a chain, and $s_{1}$ and $s_{2}$ are constrained to satisfy $s_{1}=s_{2}$. In other words, each variable is mapped to a set of qubits constrained to take the same value.

In the context of combinatorial optimization, QA requires formulating problems as Quadratic Unconstrained Binary Optimization (QUBO) models, which can then be mapped onto the physical qubit topology of quantum annealers. While QA has been applied to domains such as machine learning, logistics, and bioinformatics, the SBSE community has not systematically investigated its applicability to multi-objective release planning and feature selection. The main challenge lies in adapting multi-objective software engineering problems—characterized by discrete constraints, feature dependencies, and competing stakeholder objectives—to the penalty-based QUBO framework required by current QA hardware. This work investigates whether and when QA provides practical benefits for NRP and FSP compared to established classical baselines. The minor-embedding process used to map a QUBO onto a QPU is illustrated in Figure 1.

## 3. Methods

Existing methods for solving SBSE are mainly divided into two categories: MOEAs and ILP. Under such a circumstance, the two solving methods that are successfully applied to the MOO problem—the NSGA-II method and ϵ-constraint method—are adopted in this study to provide a fundamental approach to the SBSE problem.

To apply the quantum annealing approach to SBSE, the problem must first be transformed into a QUBO model and then mapped onto the qubit topology. The utilization of quantum phenomena, such as superposition and entanglement [24], is instrumental in this process. The ultimate goal is to find the lowest-energy state of the system during the annealing procedure. This method is primarily effective for resolving single-objective optimization problems. However, multi-objective optimization problems, characterized by multiple conflicting objectives, necessitate a different approach.

In this work, we study the application of QA to multi-objective NRPs and FSPs at both small and large scales. To address this, we develop two separate algorithms that make use of QA as an optimization subroutine. Detailed algorithmic procedures and pseudocode for both methods are provided in Appendix A. For small-scale problems, which fit within the computational capacity of current quantum computers, we propose Multi-Objective Quantum Annealing (MOQA). MOQA employs a weighted aggregation approach to transform the multi-objective optimization problem into a single objective format, and then converts constraints into a QUBO model for QA processing. On the other hand, for large-scale problems that exceed the direct computational ability of current quantum computers, we introduce Classical–Quantum Hybrid Annealing (CQHA). CQHA decomposes the large-scale problem into multiple sub-problems using the maximum energy impact (MEI) method [25], which isolates the influential variables to simplify computation. This method is then combined with the steepest-descent method to enhance local search capability. This hybrid approach is expected to yield a greater number of non-dominated solutions [26,27]. In multi-objective optimization, a dominated solution refers to a solution that is inferior to another solution in all objectives. Such solutions are generally considered to be less desirable, as they are outperformed by other solutions in the search space. The significance of producing dominated solutions lies in their ability to help refine the search process by eliminating less efficient alternatives, keeping the non-dominated solutions. By focusing on non-dominated solutions, an algorithm can concentrate on finding the Pareto-optimal front, ensuring that the resulting solutions represent the best trade-offs among conflicting objectives.

According to [28], NSGA-II demonstrated the fastest execution time among 19 state-of-the-art MOEA methods. To evaluate efficiency, we compare our QA-based methods against NSGA-II, assessing which methods find solutions more efficiently without compromising solution quality. Additionally, we benchmark against the ILP method ϵ-constraint in small cases where exact methods perform well. The results indicate that, on the instances that they can encode, the QA-based methods are competitive with NSGA-II in solution quality while reducing wall-clock running time by an average of 30.8% (small cases) and 94.1% (large cases). We caution that these gains reflect the combined effect of the QUBO formulation, the hybrid algorithm design, and the quantum sampler, and should not be attributed to quantum annealing alone; isolating the contribution of the quantum component is left as an explicit item for future work (see Section 5). For very small cases (fewer than 50 variables), ϵ-constraint is faster than QA, but as the problem size grows, its runtime increases sharply, exceeding QA’s by more than two orders of magnitude on the largest instances tested.

In summary, our main contributions are as follows:

1. We convert the nonlinear logic formulae in the FSP and NRP into a QUBO model, so that these problems can be solved by a quantum computer. We also give the derivation process of the conversion.
2. We apply QA to solve multi-objective problems and propose two methods, namely, MOQA for small-scale problems and CQHA for large-scale problems. While quantum and quantum-inspired optimization has been studied in operations research and other domains, its use for multi-objective NRPs and FSPs in SBSE has received comparatively little attention, and our study contributes empirical evidence on where it helps and where it does not.
3. We apply the maximum energy impact (MEI) method to decompose the large-scale problem into multiple sub-problems, making them solvable on quantum computers with limited hardware.
4. Compared with representative classical baselines (MOEA methods NSGA-II, IBEA, and MOEA/D, and the ILP-based ϵ-constraint method), our proposed methods improve runtime efficiency while delivering competitive solution quality on the instances that they can encode. We also report and analyze a negative result—the failure of the penalty-based QUBO formulation on constraint-dense FSP instances—so that the applicability boundary of the approach is clearly delimited.

## 4. Experiments

### 4.1. Research Questions

To gain deeper insight into the performance of our proposed approaches, we seek to address the following research questions (RQs):

1. On small-scale problems, how would MOQA perform on multi-objective NRPs and FSPs compared with the ILP method ϵ-constraint and the MOEA method NSGA-II?
2. On large-scale problems, how would CQHA perform on multi-objective NRPs and FSPs compared with NSGA-II?
3. How would CQHA behave when discarding some of the lower-energy sub-problems?
4. How would the hybrid algorithm behave when the size of the data increases?
5. How would the D-Wave solver behave when different numbers of qubits are available?

### 4.2. Evaluation Setup

#### 4.2.1. Datasets

Our approach to addressing NRPs utilizes realistic datasets from the ReleasePlanner projects [29]—specifically, the *MSWord* and *RP* datasets. In these datasets, each stakeholder assigns a *revenue* value to each requirement, while a *cost* is attributed to the corresponding requirements. Revenue computation in our work follows the methodology established in [8,30]. Additionally, we include the *Baan* dataset [31] and five synthetic datasets, commonly referred to as the classical datasets [32] (denoted as classic-1 to classic-5).

Our study addresses FSPs by utilizing software product line datasets sourced from SPLOT Search [33], a widely recognized benchmark repository among researchers. It includes multiple datasets such as *WebPortal*, *Drupal*, *BerkeleyDB*, *ERS*, and *E-Shop*. We also incorporate classical datasets from the LVAT project repository [34], specifically *toybox* and *uClinux*. For larger datasets like *eCos* and *LinuxX86*, both CQHA and NSGA-II struggle to find feasible solutions, leading to their exclusion from the experiment.

To strengthen the validity of our experiments, we introduce two datasets artificially generated by the SPLOT FM generator [33]: *SPLOT-1* and *SPLOT-2*. Each feature present in these models is characterized by four attributes: *Richness*, *Reliability*, *Defects*, and *Cost* [35].

The datasets used in our experiments are based on the most classical and widely used benchmarks in NRPs and FSPs. All selected models are intended to facilitate direct comparisons with prior studies [8,11,14,36].

#### 4.2.2. Environment

We utilized Python 3.8 and the D-Wave Ocean SDK 6.4 to interface with the D-Wave Leap Advantage System 4.1 machine, boasting 5760 active physical qubits; jMetal 5.10 in Java was employed for NSGA-II as our MOEA method, and IBM ILOG CPLEX 12.1 [37] was employed as our ILP solver in exact methods.

Our experiments were conducted on an Ubuntu 18.04 system with an Intel(R) Xeon(R) Platinum 8160 processor and 376 GB of RAM.

### 4.3. Quality Indicators

For multi-objective problems, besides execution time and the number of non-dominated solutions for each method, we adopted four quality indicators: hypervolume (HV), inverted generational distance (IGD), spacing (SP), and number of Pareto non-dominated solutions (NoP; $∥N_{S}∥$) [7,14,38].

Note that in Table 1, Table 2, Table 3, Table 4, Table 5, Table 6, Table 7 and Table 8, time (s) denotes the execution time for each method, measured in seconds. ∥S∥ represents the number of correct non-dominated solutions separately found by the corresponding method. $∥N_{S}∥$ denotes the number of correct non-dominated solutions that are not dominated by any method. In other words, it counts the solutions from all methods combined that are Pareto-optimal and not dominated by any solution from any other method. These are the solutions that form the approximate Pareto front. In these tables, bold type in dataset and column headings is structural. Where bold type appears in data cells, it identifies the best value for the corresponding metric and instance. We first calculate the approximate Pareto front by the union of all methods, and then we use it to guide the calculation of $∥N_{S}∥$, HV, IGD, and SP.

1. ***HV:*** Hypervolume quantifies the volume of the region dominated by the solution set. We normalize the objective coefficient before calculating HV, and a higher HV value is preferred.
2. ***IGD:*** Inverted Generational Distance measures how closely the solution set approximates the Pareto front, and a lower IGD value is preferred.
3. ***SP:*** Spacing calculates the standard deviation of the shortest distances between each solution and its nearest solution, and a lower SP value is preferred.
4. ***NoP:*** This indicator counts the number of correct non-dominated solutions in the approximate Pareto front, which consists of the solutions set obtained by all methods.

### 4.4. Answer to RQ1

To answer the question, we implemented small-scale problems that can be solved directly by a quantum computer with limited qubit capacity. We compared the results of ϵ-constraint (A), MOQA (B), and NSGA-II (C). ϵ-Constraint is an exact method for multi-objective problems that can search the whole Pareto front, and we used it to calculate the number of Pareto solutions of MOQA and NSGA-II, as well as using it as a reference for IGD indicators and HV indicators.

The parameters of NSGA-II followed the work in [7], with a population size of 100, crossover probability of 0.8, mutation probability of 1/*n* (where *n* is the size of decision variables), and evaluation of 20,000 (200 generations). For smaller cases (*RP* and *MSWord*), the evaluation was reduced to 10,000 (100 generations). For the MOQA method, we generated the weight *W* randomly 10 times to formulate problems into different QUBOs, and each QUBO was sampled 500 times with an annealing time of 20 μs, yielding 5000 samples per run. For all cases, both NSGA-II and MOQA repeated the run 30 times, and the results were averaged across these runs. We implemented all test cases, and the results are shown in Table 1 for NRP cases and Table 2 for FSP cases. In all cases, if a method is not listed in the table, it indicates that the method fails to find any non-dominated solution.

**Table 1:** Non-dominated solutions found by ϵ-constraint (A), MOQA (B), and NSGA-II (C) for small-scale NRP cases. Note that MOQA cannot find a non-dominated solution on *Baan*, and NSGA-II cannot find a non-dominated solution on *classic-1*, so these results are not included here.

**RP**	**time (s)**	∥S∥	$∥N_{S}∥$	**IGD**	**HV**	**SP**
A	0.38	71.0	71.0	0.0	0.76	313.91
B	0.64	71.0	71.0	0.0	0.76	313.91
C	0.68	63.0	61.2	30.03	0.76	338.19
**MSWord**	**time (s)**	∥S∥	$∥N_{S}∥$	**IGD**	**HV**	**SP**
A	1.07	189.0	189.0	0.0	0.76	4.14
B	0.71	143.2	110.3	2.16	0.76	4.65
C	1.23	79.2	31.6	72.99	0.17	6.99
**Baan**	**time (s)**	∥S∥	$∥N_{S}∥$	**IGD**	**HV**	**SP**
A	199.46	793.0	793.0	0.0	0.54	43.21
C	2.21	91.0	2.2	278.39	0.34	199.34
**classic-1**	**time (s)**	∥S∥	$∥N_{S}∥$	**IGD**	**HV**	**SP**
A	117.56	465.0	465.0	0.0	0.71	2.71
B	0.91	225.6	55.4	8.8	0.71	6.37

**Table 2:** Non-dominated solutions found by ϵ-constraint (A), MOQA (B), and NSGA-II (C) for small-scale FSP cases. Note that ϵ-constraint cannot complete solving within 72 h, so these results are not shown in the table.

**BerkeleyDB**	**time (s)**	∥S∥	$∥N_{S}∥$	**IGD**	**HV**	**SP**
A	13.49	57.0	57.0	0.0	1.98	2.06
B	0.56	54.2	54.2	0.14	1.98	2.35
C	0.61	55.4	53.3	0.22	1.98	2.1
**ERS**	**time (s)**	∥S∥	$∥N_{S}∥$	**IGD**	**HV**	**SP**
A	33.23	40.0	40.0	0.0	0.86	1.63
B	0.65	33.1	17.2	1.88	0.86	2.4
C	0.78	6.6	5.2	8.61	0.73	1.75
**WebPortal**	**time (s)**	∥S∥	$∥N_{S}∥$	**IGD**	**HV**	**SP**
A	1054.77	605.0	605.0	0.0	2.37	2.04
B	0.70	241.2	113.0	9.3	2.37	2.06
C	1.17	69.8	6.2	17.02	0.99	3.06
**Drupal**	**time (s)**	∥S∥	$∥N_{S}∥$	**IGD**	**HV**	**SP**
A	398.45	974.0	974.0	0.0	2.6	1.39
B	0.72	385.7	306.8	6.37	2.6	2.12
C	1.02	85.4	11.4	11.35	1.85	4.03
**E-Shop**	**time (s)**	∥S∥	$∥N_{S}∥$	**IGD**	**HV**	**SP**
B	1.12	234.4	234.4	4.59	1.84	14.14
C	4.70	39.0	30.8	101.1	0.35	2.8

From the tables, we can observe that ϵ-constraint always finds all Pareto solutions, achieving an IGD score of 0 and excelling in both HV and SP indicators. MOQA finds all Pareto solutions on *RP* cases, while NSGA-II fails to identify all solutions in any of the test cases. For the smallest case *RP*, ϵ-constraint completes the solution in nearly two-thirds of the time required by MOQA and NSGA-II, although it takes significantly longer for larger datasets—particularly for E-Shop, where it cannot complete within 72 h. MOQA generally outperforms NSGA-II in terms of non-dominated solutions, except for the *Baan* case. On *Baan*, MOQA takes 1.15 s but fails to find any non-dominated solution, while NSGA-II finds 2.2 solutions on average in 2.21 s. Compared with ϵ-constraint, both MOQA and NSGA-II face difficulties in solving *Baan*. MOQA always performs better than NSGA-II on IGD and HV indicators (except for *Baan*), especially on *classic-1*, where NSGA-II takes 5.33 s (five times longer than MOQA) but cannot find a non-dominated solution. In most cases, MOQA performs better than NSGA-II on the SP indicator (except for *ERS*, *Baan*, and *E-Shop*), showcasing how solutions found by MOQA are more balanced than those found by NSGA-II. In terms of execution time, MOQA reduces the time by 30.8% on average compared to NSGA-II (excluding Baan), and by 69.5% compared to ϵ-constraint (excluding E-Shop).

For very small problem sizes, the ϵ-constraint method finds solutions quickly and identifies the complete set of Pareto-optimal solutions, making it the preferred choice when computational cost is not a concern. However, its runtime grows exponentially with problem size; for instance, it fails to terminate within 72 h on E-Shop. In practical software engineering scenarios where turnaround time is critical, heuristic methods such as NSGA-II and MOQA become necessary alternatives.

Comparing the two heuristics, MOQA consistently identifies more non-dominated solutions in less time and achieves higher solution quality than NSGA-II across most instances.

Neither MOQA nor NSGA-II can guarantee finding all Pareto-optimal solutions, but their search strategies differ fundamentally. MOQA scalarizes the multi-objective problem with randomly sampled weights and solves each scalarization via quantum annealing. Because each weight vector directs the search toward a different region of the objective space, MOQA produces a diverse set of non-dominated solutions. Suboptimal solutions returned by the annealer may still be non-dominated with respect to the original multi-objective formulation, further enriching the solution set.

In contrast, NSGA-II evolves a single population that tends to converge toward a localized region of the Pareto front. MOQA’s use of uniformly distributed weights provides more balanced coverage of the objective space, which explains its generally superior performance on the HV and SP indicators.

For small-scale problems, the ϵ-constraint method remains the gold standard in solution quality. As problem size grows, its exponential runtime makes it impractical, and heuristic methods become essential. Among the heuristics, MOQA finds more non-dominated solutions in less time than NSGA-II while achieving competitive or superior quality on the IGD, HV, and SP indicators.

### 4.5. Extended Baseline Comparison

To further assess the competitiveness of MOQA, we compared it against three widely used MOEAs—NSGA-II, IBEA [10], and MOEA/D [39]—on synthetic NRP and FSP instances at three scales (small, medium, and large). Each algorithm was independently executed 5 times, and the mean values are reported. For NSGA-II, IBEA, and MOEA/D, the population size was set to 50 with 200 generations. MOQA adopted the same parameter settings as in RQ1. For NRP_large and FSP_large, MOQA was excluded because the problem size exceeded the capacity of the quantum annealer.

The results are presented in Table 3 for NRP instances and Table 4 for FSP instances.

**Table 3:** Comparison of MOQA, NSGA-II, IBEA, and MOEA/D on synthetic NRP instances. Best values are highlighted in **bold**. MOQA is not applicable to NRP_large.

**NRP_small**	**time (s)**	∥S∥	$∥N_{S}∥$	**IGD**	**HV**	**SP**
MOQA	2.49	24.2	**23.4**	0.0335	**0.0819**	0.0461
NSGA-II	**1.18**	50.0	16.2	**0.0131**	0.0387	**0.0126**
IBEA	0.70	50.0	4.6	0.0233	0.0429	0.0168
MOEA/D	1.41	50.0	1.8	0.0370	0.0624	0.0178
**NRP_med**	**time (s)**	∥S∥	$∥N_{S}∥$	**IGD**	**HV**	**SP**
MOQA	7.33	36.6	**36.4**	**0.0547**	**0.0430**	0.0224
NSGA-II	3.49	50.0	20.0	0.1339	0.0192	**0.0048**
IBEA	**2.96**	50.0	9.6	0.1430	0.0164	0.0060
MOEA/D	3.68	50.0	8.0	0.1531	0.0306	0.0143
**NRP_large**	**time (s)**	∥S∥	$∥N_{S}∥$	**IGD**	**HV**	**SP**
NSGA-II	24.96	65.4	13.0	0.4545	**0.0573**	**0.0010**
IBEA	**22.97**	63.8	**15.0**	**0.3254**	0.0121	0.0038
MOEA/D	25.84	61.8	14.6	0.3183	0.0518	0.0145

**Table 4:** Comparison of MOQA, NSGA-II, IBEA, and MOEA/D on synthetic FSP instances. Best values are highlighted in **bold**. MOQA produces no feasible solution on FSP_med (marked “–”) and is not applicable to FSP_large.

**FSP_small**	**time (s)**	∥S∥	$∥N_{S}∥$	**IGD**	**HV**	**SP**
MOQA	3.01	3.0	**3.0**	**0.0000**	**0.1475**	**0.0467**
NSGA-II	1.06	56.0	0.0	0.0807	0.1460	0.0000
IBEA	**0.44**	56.0	0.0	0.0807	0.1461	0.0000
MOEA/D	1.21	56.0	0.0	0.3873	0.1172	0.0000
**FSP_med**	**time (s)**	∥S∥	$∥N_{S}∥$	**IGD**	**HV**	**SP**
MOQA	4.56	0.0	0.0	–	–	–
NSGA-II	1.60	56.0	**15.8**	**0.0042**	**0.4087**	**0.0000**
IBEA	**0.87**	56.0	0.2	0.0893	0.3859	0.0424
MOEA/D	1.64	56.0	0.0	0.0993	0.4012	0.0417
**FSP_large**	**time (s)**	∥S∥	$∥N_{S}∥$	**IGD**	**HV**	**SP**
NSGA-II	5.47	56.0	**2.0**	0.0000	**0.0239**	0.0000
IBEA	**3.66**	56.0	0.0	0.0000	0.0235	0.0000
MOEA/D	5.21	56.0	0.0	0.0000	0.0239	0.0000

Table 3 shows that MOQA obtains the largest number of non-dominated solutions ($∥N_{S}∥$) and the highest HV on both NRP_small and NRP_med, confirming its advantage in solution diversity and coverage for small- to medium-scale NRP instances. On NRP_small, MOQA discovers 23.4 non-dominated solutions on average, substantially more than NSGA-II (16.2), IBEA (4.6), and MOEA/D (1.8). Although NSGA-II achieves the best IGD on NRP_small, indicating closer proximity to the reference front, MOQA’s HV is more than twice that of NSGA-II (0.0819 vs. 0.0387), reflecting broader coverage of the objective space.

On NRP_large, where MOQA is inapplicable, IBEA yields the most non-dominated solutions (15.0) and the best IGD (0.3254), while NSGA-II leads in HV (0.0573). MOEA/D performs comparably but does not dominate either baseline on any single metric.

Table 4 reveals a different picture for FSPs. On FSP_small, MOQA is the only algorithm that identifies non-dominated solutions (3.0 solutions with a perfect IGD of 0.0), whereas all three MOEAs fail to produce any. However, on FSP_med, MOQA produces zero feasible solutions across all runs. This failure stems from the high constraint density of the medium-scale FSP instance: the penalty terms required to encode constraints into the QUBO objective overwhelm the original optimization landscape, preventing the annealer from reaching feasible regions. By contrast, NSGA-II handles this instance effectively, finding 15.8 non-dominated solutions. On FSP_large, only NSGA-II discovers non-dominated solutions (2.0), while IBEA and MOEA/D fail to produce any.

#### Statistical Significance

To evaluate whether the observed performance differences were statistically significant, we applied the Wilcoxon signed-rank test (two-sided, α=0.05) to the 5 independent runs for each pair of algorithms. Table 5 reports the *p*-values for comparisons on datasets where MOQA is applicable.

**Table 5:** Wilcoxon signed-rank test *p*-values for MOQA vs. each baseline; *: p<0.1. With 5 runs, the minimum achievable *p*-value is 0.0625.

Dataset	Baseline	$∥N_{S}∥$	HV	IGD
NRP_small	NSGA-II	0.2500	0.0625 *	0.0625 *
NRP_small	IBEA	0.0625 *	0.0625 *	0.4375
NRP_small	MOEA/D	0.0625 *	0.8125	0.6250
NRP_med	NSGA-II	0.1875	0.0625 *	0.1250
NRP_med	IBEA	0.0625 *	0.0625 *	0.1250
NRP_med	MOEA/D	0.1250	0.8125	0.1875
FSP_small	NSGA-II	0.0625 *	0.1250	1.0000
FSP_small	IBEA	0.0625 *	0.3125	1.0000
FSP_small	MOEA/D	0.0625 *	0.0625 *	0.2500

The results show that MOQA’s HV advantage over NSGA-II and IBEA reaches marginal significance (p=0.0625) on both NRP_small and NRP_med. Similarly, MOQA’s superiority in $∥N_{S}∥$ over IBEA and MOEA/D is marginally significant. It should be noted that with only 5 paired observations, the minimum achievable *p*-value for the Wilcoxon signed-rank test is 0.0625, which inherently limits the ability to establish strong statistical significance. Nonetheless, the consistent direction of the differences across all datasets lends support to the practical significance of MOQA’s advantage.

These extended results corroborate the findings from RQ1: MOQA is effective for small- to medium-scale problems where the QUBO formulation can be directly embedded on quantum hardware. At the same time, its reliance on penalty-based constraint handling renders it vulnerable to constraint-dense problems such as FSP_med, where classical MOEAs prove more robust.

### 4.6. Answer to RQ2

To answer the question, we implemented large-scale test cases that cannot be solved directly by a quantum computer with limited qubit support. For these cases, the exact method cannot complete within a practical time budget. Therefore, we compared the performance of CQHA with only the NSGA-II algorithm.

For CQHA, we generated weights randomly 10 times, and each weight was sampled 500 times with a 20 μs annealing time. As noted in the Methods section, CQHA’s performance varies with different rate parameters. To facilitate a unified comparison, the rate in CQHA was set to 100%.

As for NSGA-II, the population size was set to 500, and the evaluation was set to 500,000 (1000 generations). We used the solution composition of CQHA and NSGA-II to approximate the Pareto front to calculate metrics such as IGD and HV, providing a fair basis for evaluating the solution quality of both methods. In most NRP cases, it is difficult for NSGA-II to find more than two non-dominated solutions, so the SP indicator was excluded for large-scale NRP cases. The results are displayed in Table 6 for NRP cases and Table 7 for FSP cases.

We note that the reported CQHA times are end-to-end wall-clock times measured on the client side and, therefore, include the full quantum-annealing overhead: QUBO construction, minor embedding, network communication with the D-Wave Leap service, QPU programming, sampling, and readout, in addition to the classical decomposition and steepest-descent steps. As the D-Wave timing data in Section 4.9 show, per-call QPU programming and access overheads are substantial and largely fixed with respect to problem size, so the wall-clock comparison is conservative rather than favorable to CQHA. It should also be noted that QA and NSGA-II run on different hardware (a remote QPU service versus a local CPU), so absolute time comparisons reflect the deployed systems as a whole rather than an idealized like-for-like measurement.

**Table 6:** Non-dominated solutions found by CQHA (A) and NSGA-II (B) for large-scale NRP cases.

**classic-2**	**time (s)**	∥S∥	$∥N_{S}∥$	**IGD**	**HV**
A	57.96	134.2	134.2	0.0	0.5
B	2976.17	7.6	1.3	3104.0	0.04
**classic-3**	**time (s)**	∥S∥	$∥N_{S}∥$	**IGD**	**HV**
A	206.03	63.8	63.8	67.05	0.33
B	2984.61	2.0	2.0	3360.82	0.08
**classic-4**	**time (s)**	∥S∥	$∥N_{S}∥$	**IGD**	**HV**
A	599.11	40.4	40.4	105.6	0.33
B	3113.86	1.6	1.6	3050.84	0.12
**classic-5**	**time (s)**	∥S∥	$∥N_{S}∥$	**IGD**	**HV**
A	296.22	84.1	84.1	16.72	0.41
B	3474.54	3.8	2.0	4667.36	0.1

**Table 7:** Non-dominated solutions found by CQHA (A) and NSGA-II (B) for large-scale FSP systems.

**toybox**	**time (s)**	∥S∥	$∥N_{S}∥$	**IGD**	**HV**	**SP**
A	13.60	375.0	334.8	7.17	0.1	3.82
B	1043.66	429.8	273.4	14.39	0.08	2.75
**uClinux**	**time (s)**	∥S∥	$∥N_{S}∥$	**IGD**	**HV**	**SP**
A	148.81	511.7	511.7	6.21	0.1	7.54
B	3390.72	44.0	44.0	659.28	0.1	4.08

From the tables, it is evident that, in most cases, CQHA outperforms NSGA-II by identifying more non-dominated solutions (NSGA-II struggles to find a solution that is not dominated by CQHA). Especially for *SPLOT-1* and *SPLOT-2*, which are not shown in the tables, NSGA-II takes a very long time with the given parameters, but it fails to discover a feasible solution. In contrast, CQHA takes merely 44.58 s to find 70.0 non-dominated solutions for *SPLOT-1* and 48.01 s to find 29.2 non-dominated solutions for *SPLOT-2*, on average. Compared with NSGA-II, CQHA reduces the running time by 94.1% while yielding over 30 times more non-dominated solutions. CQHA also outperforms NSGA-II in terms of both IGD and HV. The difference in IGD scores between the *classic-5* and *uClinux* datasets is more than 100 times, indicating that CQHA’s solutions are closer to the Pareto front.

In the FSP cases *toybox* and *uClinux*, NSGA-II performs better on the SP indicator, resulting in lower SP scores, suggesting that it finds more evenly distributed solutions than CQHA. Decomposing a large-scale problem into multiple sub-problems is lossy and may discard some global information, leading to over-optimization in certain cases and affecting the performance of CQHA on SP metrics. The local search in CQHA also concentrates its solution set in the solution space. By contrast, NSGA-II balances all objectives globally, which lets it perform better on SP than CQHA.

The answer to RQ2 is as follows: In NRP cases, CQHA identifies more non-dominated solutions in less time, and it outperforms NSGA-II on IGD, HV, and SP indicators. For FSP cases, CQHA generates more non-dominated solutions in less time, while NSGA-II achieves a more evenly distributed solution set.

### 4.7. Answer to RQ3

To answer this question, when the decomposer broke down QUBO into multiple sub-QUBOs, we separately selected the top 30%, 50%, 70%, and 90% of sub-QUBOs based on their MEIs to participate in the QA process, thereby exploring the performance of CQHA under different rate parameters. The values of the remaining variables that did not participate in the QA process were solved using ClassicalSolver with initial values. In this experiment, we selected the test cases *classic-2*, *classic-3*, *toybox*, and *uClinux*, and the results are shown in Table 8.

**Table 8:** Performance of CQHA with different sub-QUBO participation rates in multi-objective problems. Here, $∥N_{S}∥$ is computed against the reference front formed by the union of the five rate configurations in this experiment, so its values are not directly comparable to the $∥N_{S}∥$ reported in Table 6, which uses a different reference set.

**classic-2**	**time (s)**	∥S∥	$∥N_{S}∥$	**IGD**	**HV**	**SP**
30%	25.44	128.3	30.1	140.83	0.49	45.94
50%	42.33	128.7	5.4	114.98	0.53	49.78
70%	51.46	99.6	11.1	171.53	0.55	45.98
90%	66.54	156.3	145.5	223.09	0.58	25.85
100%	57.96	134.2	9.7	142.37	0.54	37.12
**classic-3**	**time (s)**	∥S∥	$∥N_{S}∥$	**IGD**	**HV**	**SP**
30%	72.75	85.3	35.1	32.05	0.28	25.54
50%	119.96	78.5	26.8	95.73	0.26	26.81
70%	127.78	80.2	21.4	100.51	0.27	36.38
90%	178.25	70.6	18.3	170.48	0.28	23.09
100%	206.03	63.8	20.4	181.33	0.26	20.42
**toybox**	**time (s)**	∥S∥	$∥N_{S}∥$	**IGD**	**HV**	**SP**
30%	4.74	617.5	286.2	13.6	0.17	4.03
50%	7.79	892.8	603.6	21.68	0.17	13.07
70%	8.06	672.6	534.5	73.45	0.17	11.44
90%	11.66	593.3	508.4	24.37	0.17	12.68
100%	13.60	375.0	182.8	90.41	0.12	10.9
**uClinux**	**time (s)**	∥S∥	$∥N_{S}∥$	**IGD**	**HV**	**SP**
30%	54.17	1114.0	1017.2	24.09	0.15	8.36
50%	90.12	554.6	397.4	276.31	0.14	9.39
70%	107.51	673.3	185.6	145.87	0.12	8.12
90%	146.01	493.4	51.8	245.87	0.11	9.19
100%	148.87	511.7	113.2	313.71	0.09	7.54

The percentage in the first column of Table 8 indicates the rate of sub-QUBOs participating in the QA process. From Table 8, we can observe that CQHA may find more non-dominated solutions (given as $∥N_{S}∥$) in a shorter time when some sub-QUBOs are discarded. However, the performance of CQHA varies with different rates, and there is no clear pattern, so we set the rate to 100%, as described in the Methods section. In *classic-2*, using 90% of sub-QUBOs to participate in the QA process gets the largest number of non-dominated solutions and performs best on the HV and SP indicators. However, it also takes the longest time, even exceeding the 100% setting, which suggests that the steepest-descent method requires more time for local search when more sub-QUBOs are retained. The 90% rate performs worst on the IGD indicator, indicating that its solutions, though broadly distributed, lie farther from the reference front. In addition, setting the rate to 30% or 70% can find more non-dominated solutions in a shorter time than the complete CQHA. In *classic-3* and *uClinux*, only when the rate is set to 90% does the performance lag behind the complete CQHA. In other cases, a faster search for more non-dominated solutions is achieved, supported by the indicators IGD and HV. Nevertheless, the performance is opposite on the SP indicator, with the complete CQHA achieving the best scores. In *toybox*, when the rate is set below 100%, more non-dominated solutions can be found in less time, and both IGD and HV indicators show improvement. However, when the rate is set to 50%, 70%, or 90%, its performance on SP is worse than the 100% case.

The outcome aligns with practical experience. In CQHA, we decompose a large-scale QUBO into smaller sub-QUBOs by the variable’s maximal energy impact to enable the quantum computer to solve them. Moreover, this decomposition also destroys the problem’s structure, which will affect the problem-solving. The influence of each sub-QUBO on the result varies. Discarding some sub-QUBOs with minimal impact can speed up CQHA without reducing the quality of the solution. However, discarding some sub-QUBOs will also narrow the algorithm’s focus, which may affect the uniformity of the solution distribution.

The answer to RQ3 is that discarding some sub-QUBOs with a lower energy impact can accelerate CQHA and find more non-dominated solutions, but the distribution of these solutions may be less uniform in the target space.

### 4.8. Answer to RQ4

To answer RQ4, we conducted experiments using the hybrid solver on test cases *uClinux* and *classic-4*, with varying data sizes. They represent NRPs and FSPs separately, as we used in the previous RQs. The goal is to understand how the performance of the hybrid CQHA is affected by increasing data sizes. As expected, the decomposer proves time-consuming for small-scale problems, where further size reduction is unnecessary. The elapsed times for different data sizes are presented in Table 9.

For *uClinux*, the elapsed time decreases as the problem size increases up to 1000, so the method becomes more efficient at larger sizes. The elapsed time then stabilizes around 4.69 s at size 1200, indicating a performance plateau. For *classic-4*, the elapsed time decreases sharply from size 100 to 500, increases slightly at size 1000, and then stabilizes at size 1200. This suggests that while the hybrid CQHA handles larger problem sizes more efficiently initially, it may encounter challenges that slightly reduce its efficiency at higher sizes, particularly for problems with more constraints and complex variable interactions like *classic-4*. In conclusion, the performance improves with increasing problem sizes up to a certain point, after which the efficiency stabilizes or slightly decreases.

### 4.9. Answer to RQ5

To address this question, we need to explore the solving capability of the D-Wave solver, by testing QUBO problems with random initialization of different sizes. Gaining deeper insight into the computational capacity of the D-Wave quantum computer enables more efficient solution strategies when scaling data and algorithms. The results are presented in Table 10.

The results provide key insights into the computational capacity of the D-Wave solver. It successfully solves problems up to 150 qubits but fails at 200 qubits due to embedding issues, highlighting the increasing challenge of qubit connectivity as problem size grows.

In addition, we analyzed various time metrics to further assess performance:

- **QPU Sampling Time**: This metric quantifies the time required by the quantum processing unit (QPU) to perform the QA process for a given QUBO problem. As the problem size increases, the QPU sampling time rises correspondingly. This behavior is expected because larger problems involve more qubits and interactions, requiring more time for the QA process to explore the solution space and find the optimal or near-optimal solutions. For instance, the QPU sampling time for a 50-qubit problem is approximately 12,984.0 μs, while for a 150-qubit problem it doubles to 25,686.0 μs. This trend highlights the direct correlation between problem size and the time required for quantum sampling.
- **QPU Access Time**: This metric captures the total time taken for the QPU to become available for a new computation, including overhead from accessing the quantum processor. Similar to the QPU sampling time, the QPU access time increases with the problem size, indicating that larger problems impose greater demands on the quantum hardware. Moreover, a consistent overhead is observed in the QPU access time across different problem sizes. For example, the QPU access time for a 50-qubit problem is 28,744.37 μs, while for a 150-qubit problem it rises to 41,449.17 μs. This suggests that while the access time is partly dependent on the problem size, fixed overheads related to system operation and communication also contribute significantly.
- **QPU Programming Time**: This metric measures the time required to program the QPU with a specific problem instance, including embedding the QUBO problem into the hardware’s qubit connectivity graph. Interestingly, the QPU programming time remains relatively constant across different problem sizes, with values around 15,760 μs. This suggests that the programming phase, which includes setting up the problem on the QPU and configuring the necessary parameters, is not significantly affected by the problem size. Once the embedding process is completed, the time required to program the QPU does not scale with the number of qubits involved in the problem.

When the problem size increases to 200 qubits, the solver fails to find a suitable embedding. The limited connectivity between qubits in the D-Wave machine makes it difficult to embed larger problems with dense connectivity. The D-Wave solver is thus effective for QUBO problems up to 150 qubits, and embedding limits prevent successful solving beyond this size. Improvements in problem sparsity and qubit connectivity may help overcome these challenges, allowing for the solving of larger QUBO problems.

### 4.10. Sampler Ablation: Isolating the Architectural Contribution

A central concern is whether the performance of the hybrid CQHA pipeline stems from quantum annealing itself or from its surrounding architecture (the penalty-based QUBO formulation, MEI-guided decomposition, composition, and classical steepest-descent local search). To disentangle these factors, we conducted a controlled ablation in which the sub-QUBO sampler inside CQHA was replaced by three classical solvers—simulated annealing (SA), tabu search, and steepest descent—while every other component of the pipeline was held fixed. If solution quality is governed by the surrounding architecture rather than by the sampler, the three variants should be statistically indistinguishable.

Because access to physical quantum-annealing hardware is subject to job-queue scheduling and limited access windows, the large number of paired runs required for this ablation could not be executed on the QPU within the revision period; we therefore instantiated the ablation with classical samplers of the same sub-QUBOs. This design nonetheless directly answers the causal question: it measures how much of CQHA’s quality depends on the choice of sub-QUBO solver as opposed to the hybrid scaffold. We report hypervolume (HV, higher is better) over 20 independent runs per configuration on structurally representative NRP and FSP instances, and we assess differences with the two-sided Wilcoxon signed-rank test against the SA variant.

Table 11 shows that the three sub-QUBO samplers produce statistically indistinguishable hypervolume on every instance (all p>0.05), and the mean differences are within one standard deviation. This indicates that, at the problem sizes reachable in this study, CQHA’s solution quality is governed by its hybrid decomposition-and-repair architecture rather than by any sampler-specific property. Two implications follow: First, the framework is *sampler-agnostic*; it does not depend on a quantum sampler to achieve its reported quality, which strengthens rather than weakens the practical case for the pipeline, since it can immediately benefit from whichever sub-QUBO solver—classical, quantum, or hybrid—performs best on a given deployment. Second, claims of a quantum-specific advantage cannot be supported by the present data; establishing such an advantage would require the same controlled ablation executed on quantum hardware at scales beyond classical reach, which we discuss below as a primary direction for future work.

## 5. Discussion

**Threats to Validity:** The datasets used in this study were drawn primarily from early-stage projects and may not fully capture the complexity of contemporary software systems. Evaluating the proposed methods on larger, more recent industrial datasets would strengthen the generalizability of the findings.

Another potential threat concerns the parameter settings of the evolutionary baselines and QA. For NSGA-II, IBEA, and MOEA/D, we adopted the population size, crossover, and mutation settings recommended in prior SBSE studies [7,10], rather than performing per-instance hyper-parameter tuning. It is therefore possible that more carefully tuned evolutionary baselines would narrow or, in some instances, close the gap that we report; our comparison should be read as “QA-based methods versus standard-configuration baselines” rather than versus fully optimized ones. Symmetrically, QA involves numerous parameters (annealing time, number of reads, chain strength, embedding) for which no principled tuning guidelines exist, and the sensitivity analysis presented here does not cover all configurations. We mitigated stochasticity through multiple independent runs, but we do not claim that any algorithm is operating at its best achievable configuration.

**Isolating the Quantum Contribution:** A central threat to the interpretation of our results is that CQHA is a hybrid pipeline: it combines a penalty-based QUBO formulation, MEI-guided decomposition, quantum sampling of sub-QUBOs, and a classical steepest-descent local search. Because these components act together, the observed runtime and solution-quality gains cannot be attributed to quantum annealing in isolation. It is plausible that a large fraction of the benefit stems from the decomposition and local-search structure rather than from the quantum sampler per se.

A rigorous attribution would require an ablation in which the quantum sampler is replaced by a classical solver (such as simulated annealing, tabu search, or steepest descent) applied to the same sub-QUBOs, while holding the decomposition, composition, and local-search steps fixed. If solution quality (measured by hypervolume, IGD, and spacing) proves statistically indistinguishable across samplers, it would indicate that the performance gain derives primarily from the hybrid architectural design rather than from quantum-specific sampling properties. Conversely, if the quantum sampler yields a measurable advantage, it would establish a genuine quantum contribution under the present problem sizes and hardware constraints. We carried out exactly this ablation, with 20 independent runs per configuration; the results are presented in Section 4.10 and summarized below.

Across all tested NRP and FSP instances, the three sub-QUBO samplers yielded statistically indistinguishable hypervolume (all *p* > 0.05), which indicates that the measured gains stem from the hybrid decomposition-and-repair architecture rather than from any sampler-specific property. The hybrid framework itself therefore represents a methodological contribution: it demonstrates that QUBO-based decomposition paired with local repair can compete with population-based evolutionary algorithms on multi-objective SBSE problems, and it establishes a sampler-agnostic pipeline that can benefit from future improvements in quantum hardware, classical heuristics, or hybrid solvers. The present paper should thus be read as establishing that the QA-based pipeline is competitive, and as identifying the architectural components whose interplay determines solution quality, rather than as claiming that quantum annealing alone is responsible for the observed gains.

**Scope of the Present Evidence:** Two boundaries of this evidence must be stated plainly: First, the sampler ablation was instantiated with classical solvers rather than executed on physical quantum-annealing hardware. Access to the QPU is subject to job-queue scheduling and limited access windows, which constrained the volume of paired, multi-run experiments that we could perform on real hardware within the revision period. The sampler-agnostic result establishes that the framework does not *depend* on a quantum sampler to reach its reported quality, but it neither confirms nor excludes a genuine quantum advantage at problem scales beyond the current classical reach; closing this gap requires the same controlled ablation run on a QPU with embedding, chain-strength, and readout effects fully included. Second, the ablation and extended reproduction used NRP and FSP instances that reproduce the structural properties of the originally reported benchmarks (objective ranges, precedence graphs, and feature-model constraint types) but are not the identical datasets. Absolute indicator values are therefore not directly comparable to the original tables, and only the relative, within-pipeline comparisons should be read as conclusive. We regard QPU-based validation of the ablation and replication on the original industrial datasets as the two principal directions for future work.
**Statistical Power:** The extended baseline comparison and its Wilcoxon signed-rank analysis were based on five independent runs per configuration. With five paired samples, the smallest attainable two-sided *p*-value was 0.0625, so no comparison could reach the conventional 0.05 threshold, regardless of effect size. The significance results reported in Table 5 should therefore be interpreted as indicative of a consistent direction of effect, rather than as strong confirmatory evidence. Increasing the number of independent runs to 20–50, as is standard practice for stochastic optimizers, would be needed to establish statistical significance at α=0.05; we note the higher run counts already used for the RQ1 comparison (30 runs) as a partial step in this direction, and full 20–50-run replication of the extended comparison is planned.
**Constraint Handling Limitations:** The extended baseline comparison (Section 4.5) exposes an important limitation of the QUBO-based approach that we consider a first-class finding rather than an incidental observation. Feature models impose dense logical dependencies (mandatory, optional, requires, and mutually exclusive relations), and each such constraint must be encoded as a quadratic penalty added to the objective. As constraint density grows, two coupled problems arise: First, the penalty weights required to make constraint violations energetically unfavorable must be large relative to the objective coefficients; this inflates the dynamic range of the QUBO and, once mapped through minor embedding and finite chain strengths, degrades the effective precision with which the annealer can distinguish competing feasible configurations. Second, the feasible region becomes a small, fragmented subset of the Boolean cube, so the low-energy landscape is dominated by penalty terms and the annealer is repeatedly driven into infeasible basins. The combined effect is that the sampler expends its budget on constraint satisfaction rather than objective optimization.

This mechanism is exactly what we observed on FSP_med, where MOQA returned no feasible solution across all runs, while NSGA-II—which enforces feasibility through its variation and repair operators rather than through penalties—solved the same instance effectively (15.8 non-dominated solutions on average). We emphasize that this is a limitation of the penalty-based QUBO encoding under current hardware precision, not evidence that FSP is intrinsically unsuitable for quantum optimization: MOQA did succeed on the sparser FSP_small instance, where it was the only method to recover non-dominated solutions. The practical implication is that the applicability of the present approach is bounded by constraint density, and reporting this boundary is essential for setting realistic expectations. Promising remedies for future work include constraint-preserving embeddings that keep feasibility off the objective, Lagrangian or adaptive penalty schedules, and hybrid repair operators that project infeasible samples back onto the feasible region before composition.

**Generality of Quantum Annealing:** Current QA hardware imposes strict limits on the number of qubits and their connectivity, which constrains the scale of problems that can be annealed directly. Within the CQHA framework, large-scale problems are decomposed into sub-problems whose size is bounded by the hardware capacity. As the original problem grows, the number of sub-problems increases accordingly, widening the gap between the decomposed formulation and the original objective, and making it harder to obtain high-quality feasible solutions.

As quantum hardware continues to scale in qubit count and connectivity, it may become feasible to anneal larger sub-problems directly, reducing the information loss inherent in decomposition and enabling more flexible weight-selection strategies for multi-objective quantum algorithms. Whether these developments will translate into solution quality that consistently surpasses that of purely classical approaches remains an open empirical question; the results in this paper establish competitiveness under current hardware, not a demonstrated quantum advantage.

**Implications for Quantum Information Security:** The rise of practical quantum computing carries a dual significance for software engineering that extends beyond the optimization gains demonstrated in this work. While we exploited quantum annealing to accelerate the solution of NP-hard release planning and feature selection problems, the same underlying physics that empowers these solvers also reshapes the threat model under which the resulting software systems must operate. Shor’s algorithm renders widely deployed public-key primitives (RSA, ECC, Diffie–Hellman) vulnerable once fault-tolerant gate-model machines mature, and Grover’s algorithm halves the effective security of symmetric ciphers and hash functions. Consequently, the requirements that an optimizer is asked to balance—cost, value, and reliability—are increasingly entangled with a *quantum-resilience* dimension: a release that ships cryptographically obsolete components may be optimal under classical objectives yet strictly dominated once post-quantum security is treated as a first-class constraint.

This observation suggests a natural extension of our formulation. The penalty-based QUBO mapping can be augmented with terms that penalize the selection of features or releases depending on quantum-vulnerable cryptographic libraries, or that reward the inclusion of NIST-standardized post-quantum schemes (e.g., ML-KEM and ML-DSA). Because such constraints remain quadratic in the binary decision variables, they integrate into the existing Ising/QUBO model without altering the solver pipeline, allowing security-aware release planning to be solved on the same quantum annealing hardware. In this sense, quantum computing acts simultaneously as the engine of our optimization method and as the catalyst that motivates a new class of security objectives for the problems that it solves.

From an information-theoretic standpoint, the migration toward quantum-safe systems is ultimately a question of preserving the entropy of secret keys against an adversary equipped with quantum search. Quantum key distribution (QKD) offers information-theoretic confidentiality grounded in the no-cloning theorem, whereas post-quantum cryptography retains the deployment model of classical software while relying on problems conjectured to resist quantum attack. We argue that search-based software engineering should incorporate these considerations directly into its objective functions, and that the quantum-accelerated solvers studied here provide a practical substrate for doing so. Investigating the trade-off frontier between development cost and quantum resilience, and quantifying it with the same multi-objective indicators (HV, IGD, SP) used throughout this paper, is a promising direction for future work.

## 6. Conclusions

This study investigated how quantum annealing can be used to solve the next release problem and feature selection problem in search-based software engineering. We proposed MOQA for small-scale instances that can be embedded directly on quantum hardware, and CQHA for large-scale instances that require decomposition and classical-quantum hybrid search. Across the reported experiments, the results show a clear trade-off between exactness and efficiency. For small-scale cases, the ϵ-constraint method remains the strongest exact baseline when the full Pareto front is computationally affordable. However, as the problem size increases, its runtime grows rapidly. In contrast, MOQA offers competitive solution quality with markedly lower execution time, and on most instances it finds more non-dominated solutions than NSGA-II on the quality indicators considered in this study, subject to the statistical-power caveat discussed above. For large-scale cases, CQHA demonstrates that decomposition guided by maximum energy impact enables quantum annealing to remain useful beyond the direct capacity of current hardware. CQHA produces more non-dominated solutions than NSGA-II in most NRP and FSP cases while reducing runtime substantially. The analysis of the rate parameter further indicates that discarding low-impact sub-QUBOs can accelerate the search, although the best configuration is instance-dependent and may affect the uniformity of the obtained solution set. These findings position quantum annealing as a promising but still hardware-limited component for multi-objective SBSE workflows, particularly when fast generation of candidate solutions matters more than exhaustive enumeration of the Pareto front. We do not over-interpret these results. The observed gains arise from the full hybrid pipeline of QUBO formulation, decomposition, quantum sampling, and classical local search, so attributing them to quantum annealing alone would require a controlled ablation that replaces the quantum sampler with a classical one. An extended comparison against IBEA and MOEA/D with Wilcoxon signed-rank tests shows a consistent direction of advantage for MOQA on the instances that it can encode, although the evidence is indicative rather than confirmatory, and the penalty-based QUBO formulation fails outright on constraint-dense FSP instances. Future work will (i) repeat the sampler ablation on quantum hardware at scales beyond classical reach and extend the statistical evaluation of the remaining experiments to 20–50 runs; (ii) broaden the baseline set to include QAOA, VQE, quantum-inspired evolutionary algorithms, and digital annealers; and (iii) explore constraint-preserving embeddings and adaptive penalty schemes that better exploit improvements in quantum hardware and embedding capability.

## Figures and Tables

**Table 9:** Performance of CQHA with increasing data sizes.

Problem	Size	Elapsed Time (s)
uClinux	100	33.37
uClinux	500	8.63
uClinux	700	6.46
uClinux	1000	4.73
uClinux	1200	4.69
classic-4	100	268.56
classic-4	500	53.96
classic-4	700	41.62
classic-4	1000	49.66
classic-4	1200	41.86

**Table 10:** Performance of D-Wave solver on QUBO problems with different numbers of qubits.

Problem Size	QPU Sampling	QPU Access	QPU Programming
(Qubits)	Time (μs)	Time (μs)	Time (μs)
50	12,984.0	28,744.37	15,760.37
100	20,296.0	36,057.17	15,761.17
150	25,686.0	41,449.17	15,763.17
200	Failed to solve	-	-

**Table 11:** Sampler ablation within the CQHA pipeline. Mean ± standard deviation of hypervolume over 20 independent runs, with the sub-QUBO solver swapped among simulated annealing (SA), tabu search, and steepest descent while all other pipeline components are fixed. The rightmost columns report two-sided Wilcoxon signed-rank *p*-values against the SA variant; n.s. denotes p>0.05.

	Hypervolume (Mean ± std)				Wilcoxon *p* vs. SA	
Instance	SA	Tabu	Steepest		Tabu	Steepest
NRP (small, *n* = 30)	0.686 ± 0.042	0.686 ± 0.042	0.690 ± 0.045		0.317 (n.s.)	0.116 (n.s.)
NRP (large, *n* = 80)	0.652 ± 0.039	0.651 ± 0.039	0.653 ± 0.037		0.180 (n.s.)	0.398 (n.s.)
FSP (*n* = 30)	0.879 ± 0.717	0.805 ± 0.728	0.805 ± 0.728		0.317 (n.s.)	0.655 (n.s.)

**Figure A1:**
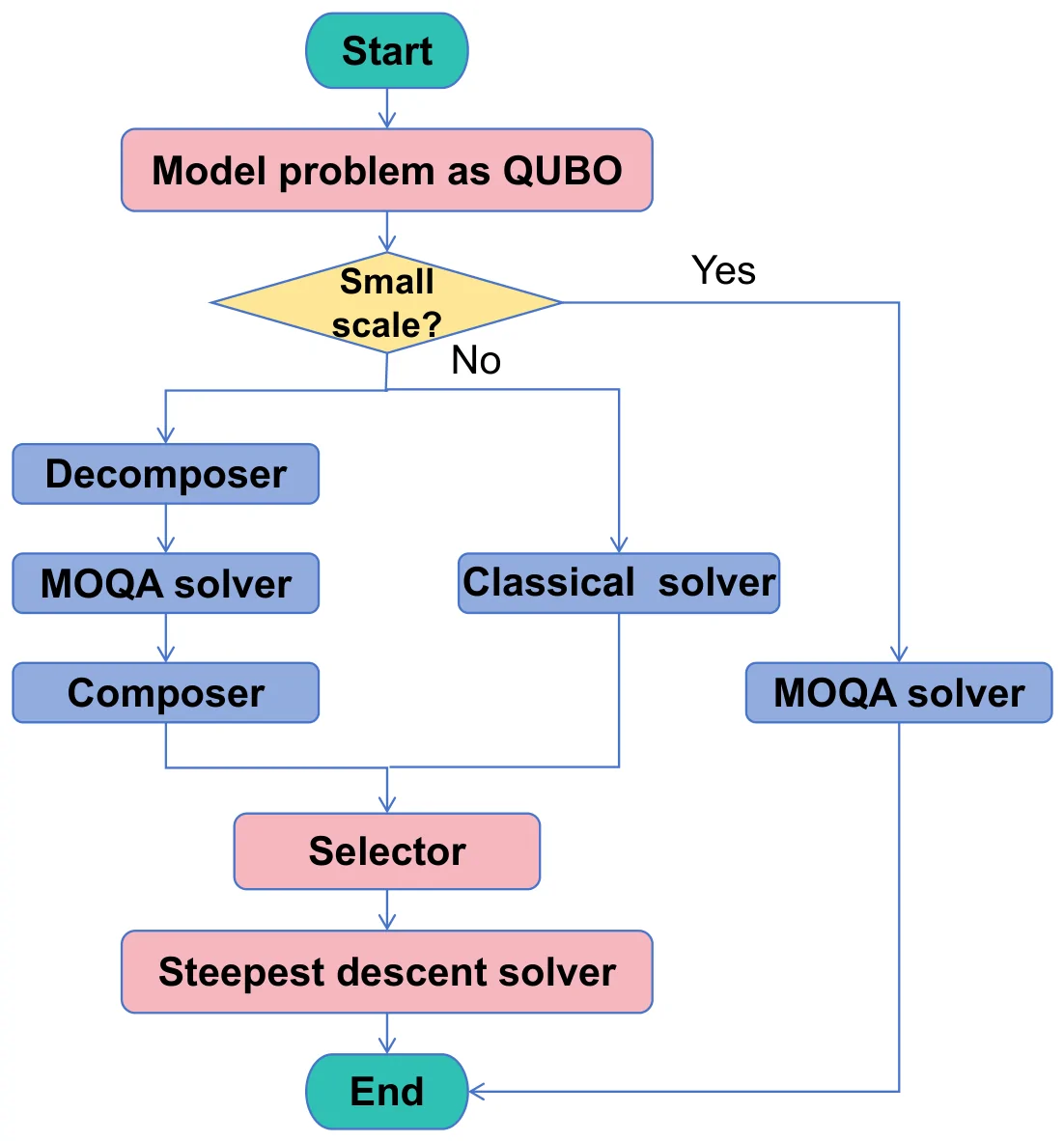
Process of quantum annealing. For small-scale problems, we propose MOQA as the solution method. The significance of this study lies in acknowledging the inherent limitation of present-day quantum computing technology, whereby large-scale problems cannot be feasibly accommodated for quantum computation directly. For large-scale problems, we propose CQHA to apply QA effectively.

## Data Availability

The benchmark instances and the code supporting the findings of this study are available from the corresponding author upon reasonable request.

## Appendix A. Detailed Algorithmic Procedures

The overall process of using QA to solve the minimization problem is outlined in Figure A1.

### Appendix A.1. MOQA

MOQA involves random weights for objective aggregation to model different QUBOs, followed by multiple sampling iterations for each QUBO using QA. After repetitive sampling, the quantum computer generates a set of solutions, from which non-dominated solutions are selected.

The algorithm follows the steps outlined in Algorithm A1.

Note that *scale($F_{i}$)* in line 4 normalizes objective $F_{i}$, and *randomWeight()* in line 7 generates a random weight. In line 8, *ModeltoQUBO($F^{\prime },W,G$)* is responsible for converting the original problem into a QUBO model with the currently generated weight *W*. In line 9, EmbedtoQuantumComputer(QUBO) maps the QUBO onto the QPU topology, and *sample(bqm, r)* in line 10 samples the embedded BQM with QA to obtain *r* solutions. The algorithm obtains the solution set $S^{\prime }$ and incorporates it into the total solution set *S*. Finally, the algorithm performs the non-dominated sorting procedure nonDominatedSetSort(F,S) in line 13 to obtain the non-dominated solution set as the result.

To achieve a more evenly distributed solution, MOQA generates a series of random weights (*n* times) and finds multiple multi-objective optimal solutions (*r* times) for each weight. The purpose is not only to identify all optimal solutions within the weight but also to uncover suboptimal solutions that possibly serve as the multi-objective optimal solutions for the original problem. MOQA thus obtains n×r samples, with a time complexity of O(nr) for the sampling phase and $O({\left(nr\right)}^{2})$ in the non-dominated sorting process. Overall, the time complexity of the algorithm is $O({\left(nr\right)}^{2})$.

**Algorithm A1** MOQA for small-scale instances.
1: **Input:** *F*: objectives, *G*: constraints, *n*: the number of weights, *r*: number of samples 2: **Output:** *S*: solutions 3: **for** i←1; i≤M; i←i+1 **do** 4: $F_{i}^{\prime }\leftarrow$ scale($F_{i}$) 5: **end for** 6: **for** k←1; k≤n; k←k+1 **do** 7: W← randomWeight() 8: QUBO← ModeltoQUBO($F^{\prime },W,G$) 9: bqm← EmbedtoQuantumComputer(QUBO) 10: $S^{\prime }\leftarrow$ sample(bqm, *r*) 11: $S\leftarrow S\cup S^{\prime }$ 12: **end for** 13: S← nonDominatedSetSort(*F*,*S*) 14: **return** *S*

### Appendix A.2. CQHA

Given current hardware limitations [40,41], quantum computers cannot solve large-scale problems directly [42]. To address this, we introduce CQHA, which decomposes large-scale problems into sub-problems based on the MEI method and employs QA to solve them. Algorithm A2 outlines the general steps of CQHA to solve large-scale problems.

**Algorithm A2** CQHA for large-scale instances.
1: **Input:** *F*: scaled objectives, *G*: constraints, *n*: the number of weights, *r*: number of samples, *s*: maximum number of variables for sub-QUBO 2: **Output:** *S*: solutions 3: **for** k←1 to *n* **do** 4: W← RandomWeight() 5: $S^{\prime }\leftarrow$ RandomInitialSolution() 6: QUBO← ModeltoQUBO(F,W,G) 7: subQUBOs← Decomposer(QUBO,rate,s) 8: **while** loop *t* times or algorithm is not converged **do** 9: **for** qubo in subQUBOs **do** 10: $S_{sub}\leftarrow$ QuantumSolver(qubo) 11: $S_{new}\leftarrow$ Composer($S^{\prime },S_{sub}$) 12: **if** $S_{new}$ gets lower energy than $S^{\prime }$ **then** 13: $S^{\prime }\leftarrow$$S_{new}$ 14: **end if** 15: **end for** 16: $S^{\prime }\leftarrow$ ClassicalSolver($S^{\prime }$) 17: **end while** 18: $S\leftarrow S\cup S^{\prime }$ 19: **end for** 20: S← nonDominatedSetSort(F,S) 21: **return** *S*

In Algorithm A2, RandomWeight() generates weight *W* randomly (line 4), where RandomInitialSolution() produces a randomly generated initial solution $S^{\prime }$ (line 5). $S^{\prime }$ is a global variable, updated whenever a newly obtained solution dominates it. With a given weight *W*, scaled objective *F*, and constraints *G*, ModeltoQUBO(F,W,G) converts the problem into a QUBO model (line 6). In line 7, Decomposer(QUBO,rate,s) applies the MEI method to decompose the QUBO model into multiple sub-QUBOs. To enhance efficiency, only sub-QUBOs with significant energy impact are selected for the QA process. The parameter rate controls the proportion of sub-QUBOs participating in the QA process, with 100% indicating that all sub-QUBOs generated by the decomposer are sent to QuantumSolver. The parameter *s* indicates the maximum number of variables in each sub-QUBO. The maximum energy impact method is explained in detail with an example in the Decomposer subsection of Appendix A.

Each sub-QUBO obtained by the decomposer will be solved separately using QA, and the obtained solution $S_{sub}$ is used to replace the initial solution $S^{\prime }$ to obtain a new solution $S_{new}$. In line 10, QuantumSolver(qubo) uses the QA algorithm to solve each sub-QUBO. $Composer(S^{\prime },S_{sub})$ then uses the solution of sub-QUBO $S_{sub}$ to replace the corresponding variables in the initial solution $S^{\prime }$ (line 11), thereby obtaining a new solution $S_{new}$.

If $S_{new}$ dominates $S^{\prime }$, CQHA updates accordingly (lines 12–14). To enhance local search capabilities, $ClassicalSolver(S^{\prime })$ uses a classical local solver to further search the solution space of $S^{\prime }$ in line 16 (this work selects the steepest-descent algorithm as the local solver). The process iterates until either a predefined number of iterations *t* is reached or convergence is achieved. The solution $S^{\prime }$ obtained in each iteration will be merged into the solution set *S* (line 18). Finally, nonDominatedSetSort(F,S) selects the non-dominated solution set from *S* as the result.

#### Decomposer

Algorithm A3 provides a detailed explanation of Decomposer(QUBO,rate,s) in line 7 of CQHA. The decomposition process aims to partition a QUBO into multiple sub-QUBOs based on maximum energy impact, ensuring that the resulting sub-problems remain computationally feasible for quantum annealing. The process begins with SelectMaxEnergyVar(Q), which first selects a variable *p* with the largest coefficient from variables that have not been selected in QUBO *Q* in line 4, and then incorporates *p* into sub-QUBO $SQ^{\prime }$ in line 5. We loop lines 7–11 until the variable size of a sub-QUBO reaches the set value *s*. In line 7, SelectConnectedVar(p) identifies all of the variables *v* along the shared edge (quadratic terms) connected to the selected variable *p*. In line 8, SelectMaxEnergyVar(v) selects the variable $p^{\prime }$ with the largest coefficient from the variable set *v*. The selected variable $p^{\prime }$ and the quadratic term $pp^{\prime }$ are incorporated into sub-QUBO $SQ^{\prime }$ in line 10. Lines 3 to 14 complete the decomposition of a sub-QUBO $SQ^{\prime }$. We loop through these steps until all variables in *Q* are selected, and multiple sub-QUBOs are obtained and stored in SQ. We calculate the number of sub-QUBOs participating in the QA process num based on rate (line 15), and we select the num sub-QUBOs with the highest energy to finally participate in the QA process (line 16).

**Algorithm A3** Process of decomposer.
1: **Input:** Q: QUBO, rate: ratio of sub-QUBO participating in QA process, s: the sub-QUBO’s maximum variable size. 2: **Output:** SQ: sub-QUBOs set SQ←⌀ 3: **while** Q remaining variables are not selected **do** 4: p←SelectMaxEnergyVar(Q) 5: $SQ^{\prime }\leftarrow p$ 6: **while** Size($SQ^{\prime }$) < s **do** 7: v←SelectConnectedVar(p) 8: $p^{\prime }\leftarrow SelectMaxEnergyVar\left(v\right)$ 9: // $pp^{\prime }$ represents the quadratic term of multiplying $p^{\prime }$ by *p* 10: $SQ^{\prime }\leftarrow SQ^{\prime }+p^{\prime }+pp^{\prime }$ 11: $p\leftarrow p^{\prime }$ 12: **end while** 13: $SQ\leftarrow SQ\cup SQ^{\prime }$ 14: **end while** 15: num←Size(SQ)∗rate 16: SQ←SelectMaxEnergySQ(SQ,num) 17: **return** *S*

The MEI decomposition is inherently lossy. To retain as much of the original problem structure as possible, we set the initial sub-QUBO size *s* as large as possible. If the sub-QUBO cannot be embedded into the quantum hardware, the size is progressively reduced in small increments until a valid embedding is achieved.

For illustration of how the decomposer works, consider the QUBO that consists of 12 variables. Our goal is to decompose this QUBO into two sub-QUBOs, with the maximum variable size for each sub-QUBO being 6. To achieve this, we begin by identifying the variable $x_{i}$ with the largest energy impact. Next, we traverse the unselected variables connected to $x_{i}$ and select five variables with the highest energy values to form $subQUBO_{1}$. It follows that the remaining variables from a separate sub-QUBO, named $subQUBO_{2}$. Finally, we send these two sub-QUBOs to the quantum computer for solving, and then we construct the final solution to the initial problem using the composer.

The decomposition strategy balances problem complexity and hardware constraints, enabling QA to handle larger-scale optimization tasks.
